# Human Hantavirus Infections, Sweden

**DOI:** 10.3201/eid0911.030275

**Published:** 2003-11

**Authors:** Gert E. Olsson, Fredrik Dalerum, Birger Hörnfeldt, Fredrik Elgh, Thomas R. Palo, Per Juto, Clas Ahlm

**Affiliations:** * Swedish University of Agricultural Sciences, Umeå, Sweden; † Umeå University, Umeå, Sweden; 1 Present address: Stockholm University, Stockholm, Sweden.; 2 Present address: Mid Sweden University, Sundsvall, Sweden.

## Abstract

The prevalent human hantavirus disease in Sweden is nephropathia epidemica, which is caused by *Puumala virus* and shed by infected bank voles (*Clethrionomys glareolus*). To evaluate temporal and spatial patterns of this disease, we studied 2,468 reported cases from a highly disease-endemic region in northern Sweden. We found that, in particular, middle-aged men living in rural dwellings near coastal areas were overrepresented. The case-patients were most often infected in late autumn, when engaged in activities near or within manmade rodent refuges. Of 862 case-patients confident about the site of virus exposure, 50% were concentrated within 5% of the study area. The incidence of nephropathia epidemica was significantly correlated with bank vole numbers within monitored rodent populations in part of the region. Understanding this relationship may help forestall future human hantavirus outbreaks.

Members of the genus *Hantavirus* (family *Bunyaviridae*) are commonly transmitted to humans through infected rodent excretions and may cause two severe human diseases: hemorrhagic fever with renal syndrome and hantavirus pulmonary syndrome (*1*,*2*). Ecologic factors that mediate hantavirus distribution and maintenance in rodent host populations are not well known, but local variations of hantavirus antibody prevalence within host populations have been observed (*3*). Approximately 60,000–150,000 humans are hospitalized because of hantavirus infections worldwide each year, and to date, no specific treatment is available (*4*). Nephropathia epidemica, the less severe form of hemorrhagic fever with renal syndrome, is found throughout Europe, especially in Fennoscandia and European Russia (*5*). Nephropathia epidemica is caused by *Puumala virus* (PUUV), carried naturally and shed by the bank vole (*Clethrionomys glareolus*) (*1*).

PUUV is the only hantavirus isolated so far in Sweden. Approximately 90% of all nephropathia epidemica cases in this country are found in the four northernmost counties, hereafter denoted as the northern region (*6*–*8*). In this area, nephropathia epidemica is, second to influenza, the most prevailing serious febrile viral infection. Incidence rates are, on average, 20 per 100,000 cases per year (*6*,*7*). In a randomized and stratified study within northern Sweden, the prevalence of PUUV-specific immunoglobulin (Ig) M antibodies was 5.4% in adult humans, implying that approximately one eighth of human PUUV infections were diagnosed and reported (*8*). During 1998, a record number of nephropathia epidemica cases were reported in Sweden; 562 were serologically confirmed (*9*). These observations suggest that in 1998 as many as 4,500 persons in the northern region may have been exposed to and infected by PUUV.

In this study, we evaluated the demographic patterns and activities in humans that are associated with the likelihood of being diagnosed with nephropathia epidemica in the northern region; examined temporal differences in the incidence of this infection to determine seasonal or periodic patterns; determined how nephropathia epidemica at the local level related to bank vole abundance in a restricted part of the region; and examined distribution of this disease within the region.

## Materials and Methods

From January 1991 to December 1998, numbers, dates of infection, and demographic details of PUUV nephropathia epidemica cases within the northern region (i.e., the counties of Norrbotten, Västerbotten, Jämtland, and Västernorrland) were obtained from laboratory reports from the Departments of Clinical Virology and Clinical Microbiology in Umeå and Boden, Sweden, respectively. Diagnosis was confirmed by the detection of IgM to PUUV with either an immunofluorescence assay or an enzyme-linked immunosorbent assay (*10*). For the remaining part of the period studied, January 1999–December 2001, numbers and dates of confirmed cases, without demographic details, were obtained from county medical officers. The distribution of cases by county, sex, and age groups was compared to the corresponding demographic data for the entire population of the region during the study period (*11*).

To characterize exposure sites and possible risk behaviors, a questionnaire was sent to 1991–1998 nephropathia epidemica case-patients concerning activity and location when they were likely exposed to PUUV. If no answer was received within 2 months, a second letter was sent; those persons who still did not respond were contacted by telephone. This complementary telephone survey was restricted to patients whose condition was diagnosed during 1997 and 1998. To determine possible biases concerning sex and age classes in the retrieved answers, compared to data regarding the total human populations on those areas, we used G tests (log-linear likelihood models) for analyses of relative frequencies of received questionnaires (*12*).

The incubation period of nephropathia epidemica ranges from 2 to 5 weeks after infection (*13*). Because of uncertainty among many patients concerning the date of infection, we pooled cases by the season of diagnosis: winter (January–March), spring (April–June), summer (July–September), and autumn (October–December). We used analysis of variance (ANOVA) to test for variation of nephropathia epidemica incidence within and between counties, years, and seasons. Where significant F-values were observed, we tested for differences within model effects by the Tukey honest significant difference test (*12*).

The bank vole–trapping data used in the analyses are publicly accessible and originate from a long-term rodent monitoring project in the county of Västerbotten (available from: URL: http://www.eg.umu.se/personal/hornfeldt_birger/bh/sidor/index2.html). The dataset consists of trapping indices obtained from biannual samplings of small mammals. Autumn bank vole abundance was estimated by snap trapping, killing and removing, in late September within a 100 x 100-km area in which 16 trapping areas were regularly distributed, according to the Swedish National Grid. In each of the 16 5 x 5-km subareas, four 1-ha plots were subject to trapping, unless they were on untrappable sites. In all, 58 of 64 possible 1-ha plots were subject to trapping. Habitats mainly comprised managed conifer forests, i.e., *Pinus* *sylvestris* and *Picea* *abies* (78%), including clearcut areas, plantation, peat bogs (18%), and agricultural land (4%) equivalent to and reflecting the general environmental composition within the main study area.

The trapping effort per sampling event was normally 150 trap-nights per 1-ha plot. Five traps were set within a 1-m radius (e.g., in runways, at guidelines, or in holes) of each of 10 permanent stations, centered and spaced 10 m apart along the diagonal of the square 1-ha plot, for three consecutive nights. Traps were checked once each morning. The average total trapping effort per sampling occasion was ≈ 8.440 trap nights. The traps were baited with unfried Polish wicks, i.e., wicks dipped in cooking oil and rolled in wheat flour (*14*), together with pieces of dried apples.

Trapping indices represent the number of voles captured per 100 trapping nights, a reflection of the relative population size on each sampling occasion. The methods and field procedures for this small mammal sampling have been described (*15*,*16*). Bank vole–trapping indices from autumn samplings, in the end of September and early October, were used as a predictor of nephropathia epidemica incidence. Human incidence data were transformed by log-normal transformation [incidence’ = log (incidence + 1)] and bank vole autumn trapping indices by arcsine transformation [trapping index’ = arcsine {(trapping index + 0.5)√0.5}] (*12*).

Only patients who declared that they were confident about the occasion and site of their exposure were included in the individual-based spatial analysis. Each such site was assigned to the nearest 10 x 10-km node within the Swedish National Grid system, using the geographic information system ArcView (ESRI, Redlands, CA). Density estimations of the distribution of these case-patients within the northern region were then calculated by fixed kernels of 95%, 75%, and 50% probabilities (*17*). The kernel method is a nonparametric statistical tool to recognize spatial patterns and areas of defined probability and estimated range for the distribution of study objects, e.g. nephropathia epidemica cases, and was most explicitly pictured by Worton (*18*) as the following: “A scaled-down probability density function, namely the kernel, is placed above each data point and the estimator is constructed by adding the *n* components. Thus, where there is a concentration of points the kernel estimate has a higher density than where there are few points. Because each kernel is a density the resulting estimate is a true probability density function itself.”

## Results

**Figure 1:**
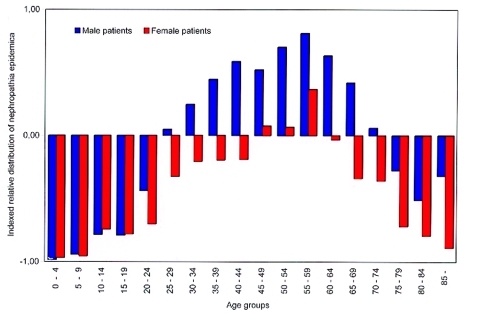
Indexed relative distribution of nephropathia epidemica (NE) cases during 1991 to 1998, within age group and sex (*i*) in relation to the total human population (pop) in the northern region (NR), where deviations from zero indicate overrepresentation vs. underrepresentation, as calculated by [index]*_i_* = [{(#NE)*_i_*½(#NE)_NR_}**½**{(#pop)*_i_*½(#pop)_NR_}–1].

From 1991 to 1998, a total of 1,724 persons were identified with serologically verified hantavirus infection (1,075 males, 649 females). We received answers to the questionnaires and telephone surveys from 1,305 persons (76%). Of these, 862 were confident about the time and location of hantavirus exposure, and information from them was used for more reliable exposure site identification. There was no bias in the received answers as compared to available human census data regarding county (G = 4.33, df = 3, p = 0.22), age group (G = 10.35, df = 13, p = 0.67, age groups <15 years and >79 years were respectively pooled) or sex (G = 0.78, df = 1, p = 0.38). Nephropathia epidemica occurred in persons of all age groups (range 3–92 years, mean age 47.4 years), but the age distribution of patients was significantly different from the mean age distribution of the entire human population in the region during the study period (G = 813.00, df = 1, p < 0.001, Figure 1). Men aged 25 to 74 years and women aged 45 to 59 years were overrepresented among the case-patients, and persons of both sexes <25 years of age were underrepresented (Figure 1). Male patients were over-represented compared to female patients (G = 103.0, df = 1, p < 0.001). Of the case-patients who answered the questionnaire and were confident about the exposure event, 82% claimed that it had occurred in or adjacent to a dwelling (year-round residence [54%] or a holiday house [28%]) and that they were engaged in handling firewood (27%), cleaning or redecorating the residence (19%), gardening or handling hay (18%).

**Figure 2:**
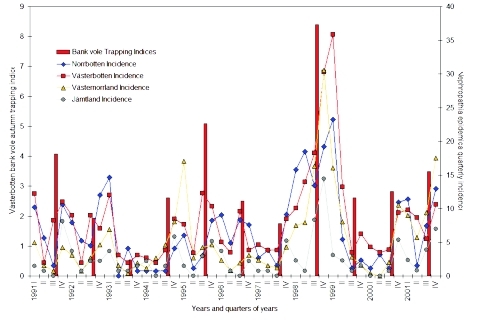
Quarterly incidence of reported nephropathia epidemica cases within respective counties of the northern region from 1991 to 2001, as represented by lines; bars represent annual autumn bank vole–trapping indices.

**Figure 3:**
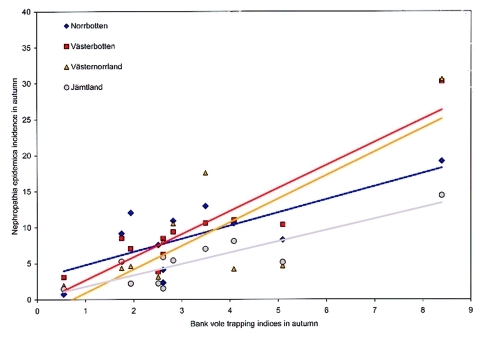
Correlation between incidence on reported nephropathia epidemica cases in respective counties within the northern region in autumn (October–December) and autumn (late September) bank vole–trapping indices in Västerbotten during 1991 to 2001 (data presented nontransformed, before statistical analyses).

**Figure 4:**
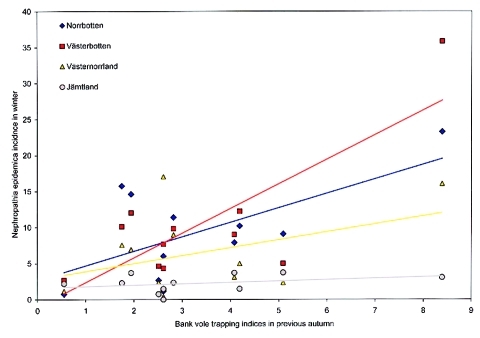
Correlation between incidence on reported nephropathia epidemica cases in respective counties within the northern region in winter (January–March) and previous autumn (late September) bank vole–trapping indices in Västerbotten during 1991 to 2001 (data presented nontransformed, before statistical analyses).

When incidence data from 1999 to 2001 were added, a total of 2,468 serologically verified nephropathia epidemica cases were identified in the northern region from January 1, 1991, to December 31, 2001, giving a yearly average incidence of 25 cases per 100,000 inhabitants during the study period (Table, Figure 2). ANOVA showed that the incidence of nephropathia epidemica was not uniformly distributed between counties (F-value = 25.9154, df = 3, p < 0.001), years (F-value = 8.3595, df = 10, p < 0.001) or seasons (F-value = 19.0528, df = 3, p < 0.001), but without interaction of counties and years (F-value = 0.8801, df = 30, p = 0.6474). Tukey tests showed that, on the county level, the inland county Jämtland (Table, Figure 2) had a significantly lower incidence than Västerbotten and Norrbotten (Tukey p < 0.05), and Västernorrland had significantly lower incidence than Västerbotten (Tukey p < 0.05). Of the 11 years included in the study, 1995, 1998, 1999, and 2001 had a higher incidence of infection than other years (Tukey p < 0.05). Of the four seasons, autumn and winter had significantly higher incidence than spring (Tukey p < 0.05), but autumn also differed from summer (Tukey p < 0.05). The seasonal periods that had significantly highest incidence, i.e., autumn and winter, were further evaluated in relation to the Västerbotten bank vole–trapping indices. We compared the preceding bank vole–sampling results of the current autumn to nephropathia epidemica incidence in autumn and winter. The highest coefficient of determination of nephropathia epidemica incidence from the bank vole index was found during autumn in Västerbotten [Västerbotten incidence’= –0.1907 + 0.8538 (trapping index’), R^2^ = 0.7526, p < 0.001], followed by Jämtland [Jämtland incidence’= –0.4046 + 0.8348 (trapping index’), R^2^ = 0.6004, p = 0.0051]; Norrbotten [Norrbotten incidence’= –0.3632 + 0.9372 (trapping index’), R^2^ = 0.5049, p = 0.0143], and Västernorrland [Västernorrland incidence’= –0.4489 + 0.9372 (trapping index’), R^2^ = 0.5049, p = 0.0143] (Figure 3). The winter period had high incidence, but the coefficients of determination between bank vole indices and incidences of nephropathia epidemica were very low and statistically significant only for Västerbotten [Västerbotten incidence’= -0.0230 + 0.6998 (trapping index’), R^2^ = 0.3753, p = 0.0451], whereas the rest were nonsignificant; Jämtland [Jämtland incidence’= 0.1733 + 0.2216 (trapping index’), R^2^ = 0.0627, p = 0.4576]; Norrbotten [Norrbotten incidence’= -0.2749 + 0.8698 (trapping index’), R^2^ = 0.3052, p = 0.0780] and Västernorrland [Västernorrland incidence’= –0.0209 + 0.5392(trapping index’), R^2^ = 0.1266, p = 0.2829] (Figure 4).

**Table:** Quarterly incidence ^a^ of nephropathia epidemica per year and county, and autumn trapping indices ^b^ of bank voles

Counties and populations		Incidence of reported nephropathia epidemica cases										
	Season	1991	1992	1993	1994	1995	1996	1997	1998	1999	2000	2001
Norrbotten	Jan–March	10.20	7.89	14.60	0.75	6.01	9.08	2.67	15.74	23.25	1.17	11.38
Inhabitants: 263.891 Inhabitants/km^2^: 3^c^	April–June	5.66	5.26	0.00	0.75	1.13	4.92	3.81	18.43	5.42	3.12	1.57
	July–Sept	1.51	4.51	4.12	0.75	3.01	8.32	1.52	13.44	1.16	1.17	7.46
	Oct–Dec	10.57	12.03	0.75	4.11	8.27	7.57	9.15	19.20	2.32	10.93	12.95
Västerbotten	Jan–March	12.21	8.98	12.01	2.69	7.68	5.00	4.63	10.09	35.84	4.30	9.81
Inhabitants: 257.860 Inhabitants/km^2^: 5	April–June	1.97	1.95	3.10	1.92	3.46	3.46	3.86	13.96	13.24	3.52	8.63
	July–Sept	8.27	8.98	1.94	3.85	12.29	9.62	3.86	18.23	3.51	3.91	5.49
	Oct–Dec	11.03	7.03	3.10	8.47	10.37	3.85	8.49	30.26	6.23	9.39	10.60
Västernorrland	Jan–March	4.98	3.07	6.91	1.15	17.04	2.34	2.36	7.54	16.04	0.41	8.98
Inhabitants: 256.777 Inhabitants/km^2^: 11	April–June	1.53	0.77	1.54	2.69	2.71	0.78	1.57	7.94	8.02	0.00	5.71
	July–Sept	0.77	2.68	0.38	4.61	4.26	1.95	1.18	16.28	2.81	2.03	9.38
	Oct–Dec	4.21	4.60	1.92	8.07	4.65	3.12	4.32	30.57	1.60	10.53	17.55
Jämtland	Jan–-March	1.47	3.68	3.67	2.20	1.48	3.72	0.75	2.28	3.06	0.00	2.33
Inhabitants: 134.324 Inhabitants/km^2^: 3	April–June	0.74	0.74	0.73	0.00	0.00	0.74	0.75	0.76	2.30	0.00	0.78
	July–Sept	0.00	2.21	0.73	1.47	2.95	0.00	0.00	8.35	0.77	0.77	3.89
No. bank voles / 100 nights^d^	Oct–Dec	8.09	2.21	1.47	5.87	5.16	2.23	5.26	14.42	1.53	5.40	7.00

^a^No. of reported nephropathia epidemica cases per 100.000 inhabitants. ^b^No. of bank voles trapped per 100 trapnights. ^c^Average population density 1991–2001 from census. ^d^Bank vole trapping indices from Västerbotten.

**Figure 5:**
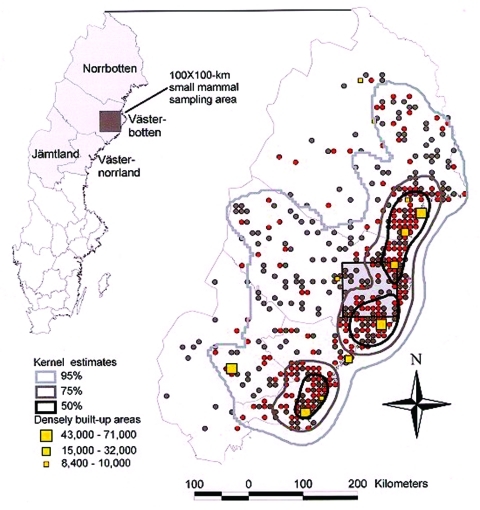
Spatial distribution of nephropathia epidemica infections during 1991 to 1998 in the northern half of Sweden as suggested by patients confident about their site of virus exposure (n = 862). Sites of virus exposure are represented by red (1998 outbreak) and gray dots (remaining years). Densely built-up areas are represented by yellow squares (8,400 to 71,000 inhabitants). Kernel estimates (bold contours) on spatial clustering of cases represent 95%, 75%, and 50% chances of encountering a case among the 862 samples.

On the fine-tuned spatial distribution, based on the 862 confident nephropathia epidemica patients, the Kernel density estimates were strongly skewed towards the coastal areas of the region (Figure 5). Ninety-five percent of the cases were found within 55% of the northern region (158,209 km^2^), the 75% Kernel covers 15% of the study area (42,762 km^2^); 50% of cases were concentrated within 5% (14,329 km^2^) of the study area.

## Discussion

We found that the persons at highest risk of having clinical nephropathia epidemica were middle-aged men, a result also observed in other European studies (*19*–*21*). However, an earlier study on a stratified and randomly selected number of people from Västerbotten and Norrbotten showed no differences between sexes in the actual prevalence of PUUV infection, and the highest prevalence of IgM antibodies was observed in persons >55 years of age (*8*). Our finding that middle-aged men were overrepresented in relation to sex and age groups, respectively, may reflect age- or sex-related difference in, for example, risk behaviors, likelihood of seeking medical attention, symptoms, or a combination of these that merit further studies.

The years of the highest incidence of nephropathia epidemica show a periodic pattern similar to that of small mammal population dynamics of northern Sweden, with approximately 3-year intervals between peaks (*15*,*16*,*22*). During these, and most other, years the incidence of this disease peaked in the autumn, with winters also showing a high incidence. However, PUUV transmission among the sexually mature bank voles, within the studied system (*23*) and in a similar endemic region (*24*), had been shown to be highest during their reproductive summer period. Within the studied populations, few newly recruited young and immature bank voles had PUUV antibodies (*23*). The temporal discrepancy on PUUV transmission between conspecific voles vs. voles to humans is likely due to bank voles’ abandoning their territoriality in autumn (*25*), with subsequent extensive individual movements and risk of human dwellings being entered and infested by infected rodents when the climate becomes more harsh (*26*,*27*). That rodents invade dwellings is also an observation made from the system of deer mouse (*Peromyscus maniculatus*) and Sin Nombre virus (*28*). This increased risk for human exposure to PUUV in autumn due to infestation of dwellings by bank voles is consistent with the fact that the within-year vole population usually peaks in autumn (*16*), when the PUUV-infected bank voles generally appear in highest numbers (*23*,*29*). The significant linear correlations of the incidence of nephropathia epidemica within the four counties to the bank vole–trapping indices from Västerbotten demonstrate a spatiotemporal synchronization for the region in bank vole dynamics. Although the strongest association was found in Västerbotten, the pattern was similar for the other counties, albeit with a lower coefficient of determination. Nevertheless, vole abundance in the autumn is a good predictor on the risk of nephropathia epidemica outbreaks during the subsequent autumn and winter. This observation is valuable since a large proportion of cases were identified adjacent to the sampling area of the long-term small mammal study. The ongoing long-term small mammal studies in the Four Corners region of the southwestern United States rapidly detected changes in population densities of the monitored rodent species, several of which were hantavirus hosts (*30*). Findings like these highlight the relevance of long-term surveys ofnatural hosts of significant zoonoses. A study during 1985 to 1991 in Västerbotten indicated a highly significant relationship between bank vole–trapping indices and nephropathia epidemica incidence during July through December (*31*). But the period January–June also showed a highly significant correlation between the number of bank voles in the previous autumn and the incidence of this disease (*32*). The same relationship, concerning the January–March period in Västerbotten, was significant in our study. However, dynamics of the local bank vole populations have changed in the sense that annual winter declines have become more precipitous (B. Hörnfeldt, pers. comm.).

We are not certain what causes the overall lower incidences in the counties of Västernorrland and Jämtland, compared to the two northernmost counties, Norrbotten and Västerbotten. A likely explanation may be a regional difference in absolute numbers of bank voles, though it is the most common small mammal species throughout the region. That the people who became infected and answered the questionnaire may or may not constitute a random sample of the population available in the respective counties with regard to behavior outside urban settlements is another factor to be considered. Any such differences may cause disparities in frequency of contacts between humans and bank voles and the subsequent risks in acquiring a PUUV infection.

Administrative limitations at the county level are inadequate to describe the actual occurrence of nephropathia epidemica in northern Sweden with any accuracy. An accurate estimate could be made by using the patterns of geographic distribution of the 862 cases (Figure 5), as described herein. Most cases in patients confident about the site of exposure were distributed along the coastal areas of the Gulf of Bothnia, where 50% of the cases clustered within 5% of the study area. This finding coincides with the actual distribution of humans in the region, and so may best explain the spatial occurrence of nephropathia epidemica. However, while the major cities and towns are located within this area, the cases are not associated with urban settlements (*8*,*32*). The remaining residents here, in rural areas, represent <30% of the total population in the northern region (*11*). The occurrence of nephropathia epidemica in relation to the actual distribution of humans within the region needs to be further evaluated to establish whether the observed pattern is merely a reflection of human population density per se, or if environmental factors also favor and promote hantavirus circulation within local bank vole populations and encounters with humans. Lack of sheltering snow cover should force bank voles to use manmade vole refuges to avoid the harsh climate and predators (*33*). Thus, the more maritime climate along the coast, in which the period of adequate snow cover is delayed and shortened in comparison to the more continental inland climate, may be a contributing factor to the regional differences in incidence (*34*).

In reply to our inquiry, persons often stated that sites of human PUUV exposure were woodsheds and woodpiles, where many may have become infected while handling firewood, which stirred up PUUV-contaminated dust particles. These peridomestic bank vole harborages provide a refuge against most predators and shelter from flooding during times of heavy rainfall. Korpela and Lähdevirta (*27*) observed a correlation between nephropathia epidemica and small rodent occurrence inside rural dwellings, particularly in cupboards, where rodent excretions were deposited. In the present study, cleaning and redecorating were among the activities reported when exposure to PUUV was thought to have occurred. These activities also put persons at risk of inhaling PUUV-contaminated dust particles.

In conclusion, middle-aged persons engaged in activities in or near manmade vole refuges were overrepresented among patients diagnosed with nephropathia epidemica. Most cases were diagnosed during autumn; this finding was more pronounced after an eruption in bank vole numbers the preceding summer and autumn. The spatial distribution of asserted sites of exposure to PUUV was skewed towards the coastal areas of the region. Bank vole dynamics and behaviors, with larger scale movements in autumn and subsequent invasion of human dwellings, together with dense human population in these areas, are among the likely candidates for the observed temporal and spatial patterns. The high human incidence and well-studied rodent community in the present system make it feasible for use as a model system to evaluate environmental factors that may influence PUUV circulation, persistence, and transmission to humans.
